# Left Atrial Ganglionated Plexi Detection is Related to Heart Rate and Early Recurrence of Atrial Fibrillation after Surgical Ablation

**DOI:** 10.21470/1678-9741-2016-0059

**Published:** 2017

**Authors:** Grzegorz Suwalski, Małgorzata M. Marczewska, Kamil Kaczejko, Jakub Mróz, Leszek Gryszko, Andrzej Cwetsch, Andrzej Skrobowski

**Affiliations:** 1 Department of Cardiac Surgery, Military Institute of Medicine, Warsaw, Poland.; 2 Department of Internal Diseases, Arterial Hypertension and Angiology, Medical University of Warsaw, Poland.; 3 Department of Cardiac Surgery, Military Institute of Medicine, Warsaw, Poland.

**Keywords:** Ganglia, Autonomic, Atrial Fibrillation, Ablation Techniques

## Abstract

**Introduction:**

Left atrial ganglionated plexi ablation is an adjuvant technique used to increase the success rate of surgical ablation of atrial fibrillation. Ganglionated plexi ablation requires previous detection. We aimed to assess determinants of successful ganglionated plexi detection and to correlate range of ganglionated plexi ablation with risk of early atrial fibrillation recurrence.

**Methods:**

The study involved 34 consecutive patients referred for surgical coronary revascularization with concomitant atrial fibrillation ablation. Ganglionated plexi detection was done by inducing vagal reflexes in the area of the pulmonary veins and left atrial fat pads.

**Results:**

Detection of GP was successful in 85% of the patients. There was no difference in preoperative characteristics nor in atrial fibrillation type between patients in whom ganglionated plexi detection was successful and others. The number of detected ganglionated plexi correlated significantly only with preoperative resting heart rate. Significant negative correlation was found in patients with preoperative heart rate>75 beat/min in terms of total number of detected ganglionated plexi (*P*=0.04). Average number of detected ganglionated plexi was significantly higher in patients with in-hospital atrial fibrillation recurrence requiring electrical cardioversion (3.8±3) in comparison to rest of the study population (2±1.3; *P*=0.02). In patients in whom 4 or more ganglionated plexi were detected, significantly increased risk of in-hospital atrial fibrillation recurrence was observed (OR 15; 95% CI 1.5-164; *P*=0.003).

**Conclusion:**

Left atrial ganglionated plexi detection was unsuccessful in a considerable percentage of patients. Preoperative heart rate significantly influenced positive ganglionated plexi detection and number of ablated ganglia. Higher number of detected ganglionated plexi was related with early recurrence of atrial fibrillation.

**Table:** 

Abbreviations, acronyms & symbols	
AF	= Atrial fibrillation
ANS	= Autonomic nervous system
GP	= Ganglionated plexi
HR	= Heart rate
LAA	= Left atrial appendage
PVs	= Pulmonary veins
SD	= Standard deviation
UFH	= Units of unfractionated heparin

## INTRODUCTION

Surgical ablation of atrial fibrillation (AF) is recommended in patients (Class IIA) undergoing cardiac surgery^[1]^. Adjunct ablation of cardiac autonomic ganglionated plexi (GP) may additionally reduce AF recurrence. However, there is no clear data on its effectiveness, though this technique was introduced a decade ago^[2]^. The only GP ablation surgical technique introduced to cardiac surgery is based on previous epicardial detection by inducing vagal reflex with rapid stimulation. The stimulation of GP results in rapid acetylcholine secretion and subsequently elicited vagal reflex (transient bradycardia, conduction block)^[3]^. Therefore, only detected GP are ablated in that technique.

Anatomical studies showed that most GP are located within the epicardial fat pads covering the entire antrum of the pulmonary veins (PVs) and the interatrial groove. There is no data showing absence of GP in certain individuals^[4]^. The success rate of detection and the number of detected GP vary in the studies published thus far, and even more limited data is available on the predictors of successful GP detection during surgical ablation of AF^[5,6]^. The primary aim of this study was to analyze the preoperative factors corresponding to successful GP detection and the number of detected GP in patients undergoing AF ablation concomitantly to surgical coronary revascularization. The secondary aim was to search for a correlation between GP detection and early recurrence of AF.

## METHODS

### Study Population

The study involved 34 consecutive patients with persistent and long-standing persistent AF and coronary artery disease referred for surgical revascularization with concomitant left atrial ablation and left atrial appendage epicardial occlusion. No inclusion or exclusion criteria were introduced for this study since all patients with AF referred to surgical revascularization are qualified for off-pump coronary artery bypass grafting with concomitant left atrial ablation and left atrial appendage occlusion in our center. All patients had a high stroke risk according to the CHA2DS2-VASc score (mean score of 3.8±1.6) as well as an increased risk of bleeding on oral anticoagulants, with an average HAS-BLED score of 3.1±1.3 (Table 1). The study protocol was approved by the Institutional Ethics Committee and all patients signed informed consent before surgery.

**Table 1:** Patient characteristics.

Parameter	Mean or number of pts.	SD or percentage
Age (years)	69.1	±7
Persistent AF	24	71%
Persistent long-lasting AF	10	29%
LA diameter (mm)	46	±5
AF duration (months)	45	±60
EuroSCORE II (%)	2.3	±1.9
Syntax Score	25.1	±7
LVEF (%)	50	±11
NYHA class	2	±0.7
Arterial hypertension	30	88%
History of MI	16	47%
Diabetes	18	53%
Vascular disease	11	32%
TE event	7	21%

AF = atrial fibrillation; LA = left atrium; LVEF = left ventricular ejection fraction; MI = myocardial infarction; NYHA = New York Heart Association; SD = Standard deviation; TE = thromboembolic event

### Surgical Procedure

**Fig. 1:**
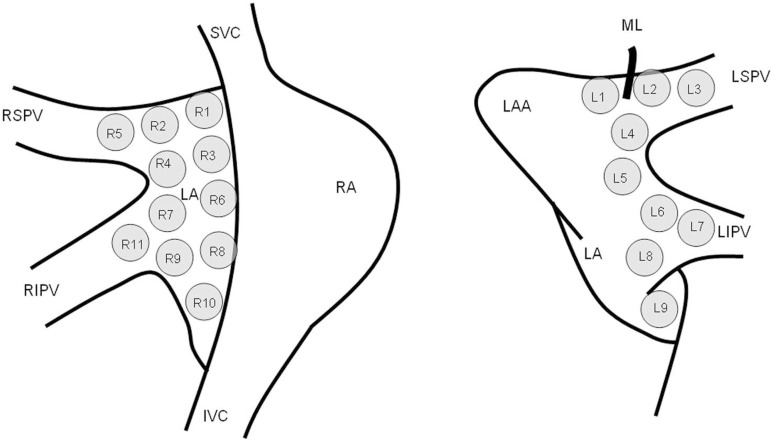
Sequence for applying rapid stimulation to detect GP. IVC=inferior vena cava; LA=left atrium; LAA=left atrial appendage; LIPV=left inferior pulmonary vein; LSPV=left superior pulmonary vein; ML=Marshall ligament; RA=right atrium; RIPV=right inferior pulmonary vein; RSPV=right superior pulmonary vein; SCV=superior vena cava.

**Fig. 2:**
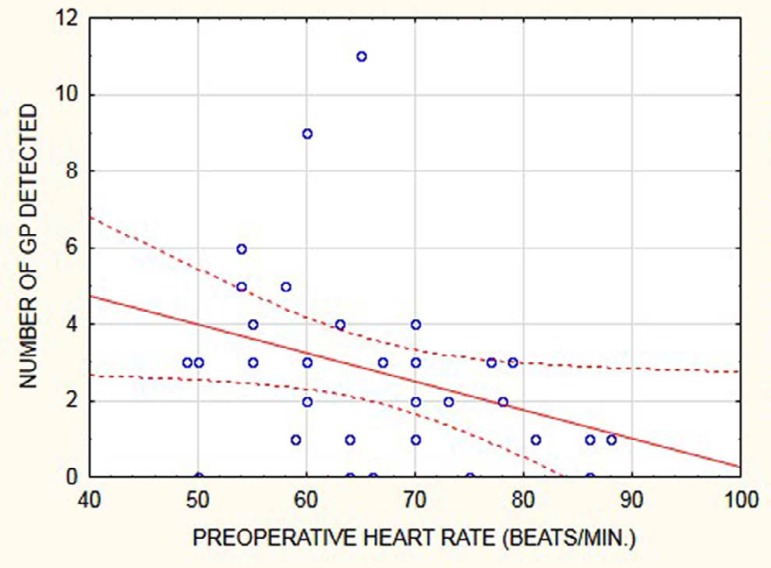
Correlation between the number of GP detected and preoperative heart rate.

In all patients, the heart was exposed with a standard median sternotomy. The transverse and oblique pericardial sinuses were dissected for full visualization of the PVs' ostia and to allow for the placement of the bipolar radiofrequency ablation device. Bilateral epicardial mapping of the PVs, left atrial antrum, left atrial appendage, and the free wall of the right atrium was done using the MAPS^®^ probe (Medtronic Inc., MN, USA) (Figure 1). Then, the first attempt at GP detection was accomplished with rapid stimulation (800 beats/min) to induce vagal reflex in the area of the PVs and left atrial fat pads. The sequence in which rapid stimulation was applied is presented in Figure 1. The pacing output started at 5 V. If detection was unsuccessful, pacing output was increased to 7 V and, finally, to 10 V. Vagal reflex (signifying GP detection) was defined as a minimum 25% reduction in heart rate or the occurrence of an atrio-ventricular conduction block. All planned mapping areas were checked in every patient. Detected GP were epicardially ablated using the same probe to apply radiofrequency energy (25 W). The ablated area was then checked with rapid stimulation for persistent vagal reflex occurrence. The PV isolation procedure was performed using a radiofrequency bipolar device (Cardioblate^®^, Medtronic Inc., MN, USA). Each ablation sequence consisted of four applications to each pair of PVs, followed by probe repositioning and another four applications (Figure 2). After each ablation sequence, mapping of PVs was carried out to check for an entrance block. This ablation sequence was repeated until no signal was detected over the PVs. Then, the Marshall fold was dissected. Patients who remained in AF were then electrically converted to sinus rhythm. The presence of an exit conduction block was assessed by PVs stimulation with a 10 V output, at a rate of 120 per minute. Next, arterial and venous grafts were harvested and the patients were heparinized with 300 units of unfractionated heparin (UFH) per kilogram. Off-pump coronary artery bypass grafting was performed. The left atrial appendage was epicardially occluded using either the AtriClip^®^ (Atricure, Dayton, OH, USA) or the Tiger Paw System II^®^ (Maquet). Completeness of left atrial appendage (LAA) occlusion was ascertained through transoesophageal echocardiography. Finally, protamine was given and the chest was closed in a standard fashion.

An amiodarone infusion was started upon admission to the intensive care unit (daily dose of 600 mg IV), continued for 48 hours, and then given as an oral dose of 200 mg once daily until hospital discharge. Starting on the 1^st^ postoperative day, all patients received bisoprolol at a daily dose of 5 mg or 7.5 mg, depending on postoperative heart rate. Electric cardioversion was performed in patients with AF recurrence, if the arrhythmia was not self-terminating within 24 hours of onset, despite a potassium level of > 4.5 mmol/l. All patients were discharged on oral amiodarone (200 mg daily for 3 months).

Early effectiveness of this ablation procedure was assessed using the composite end-point, which consisted of a need for electrical cardioversion due to in-hospital AF recurrence, the presence of AF at hospital discharge, and after 3 months. At 3 months follow up, 24-hours Holter electrocardiography study was done. AF recurrence was determined if AF episode longer than 30 seconds was recorded.

### Statistical Analysis

Statistical analysis was performed using the Statistica 12^TM^ program (StatSoft^TM^, Inc. 2012). The Shapiro-Wilk W test was used in testing for normality. If the W statistic was significant, then the hypothesis that the respective distribution is normal was rejected. Normally distributed continuous variables are expressed as mean ± standard deviation (SD). Nonparametric and parametric data correlations were evaluated with either the Spearman rank-test or the Pearson test. Comparisons of groups were performed using the Student's t-test for normally distributed continuous variables, and the Mann-Whitney test for non-normally distributed continuous variables. Differences were considered significant at *P*<0.05. Logistic regression analysis was performed to identify the risk factors associated with arrhythmia recurrence. The results are expressed as odds ratio (OR), with a 95% confidence interval (95% CI).

## RESULTS

### Successful GP Detection

Detection of GP was successful in 85% of the patients (29 patients). In 15% of the study population (5 patients), no signs of vagal reflex were observed during epicardial stimulation. The average number of detected GP was 2.7± 2.4. Significantly more GP were detected on the right side (60 in total, mean 1.7±1.5) than on the left side (31 in total, mean 0.9 ±1.2; *P*=0.01). There was no significant difference in the preoperative characteristics nor in the type of AF between patients in whom GP detection was successful *versus* those in whom it was unsuccessful (Table 2). GP were detected in 66% (n=19) of the patients with persistent AF and in all patients (n=10) with long-standing persistent AF (*P*=0.1). Detection was successful in 94% (n=18) of the patients in preoperative AF and in 75% (n=16) of the patients in sinus rhythm before surgery (*P*=0.1). In terms of diabetes, GP were detected in 78% of the diabetic patients *versus* 94% in the non-diabetic group (*P*=0.2). The occurrence of diabetes correlated, albeit insignificantly, with unsuccessful GP detection (r=-0.2251; *P*=0.2).

**Table 2:** Preoperative comparison of patients in whom GP detection was successful versus those in whom it was unsuccessful.

Parameter	Successful GP detection n=29	Unsuccessful GP detection n=5	*P* value
Age (years)	69.8±7	64.6±6	0.1
Duration of AF (months)	48±64	28.2±23	0.5
LA diameter (mm)	46.1±5	44.8±6	0.6
EuroSCORE II (%)	2.4±2	1.7±1	0.5
LVEF (%)	51.5±11	44±13	0.2
Syntax score	25±7	28±6	0.2
NYHA class	2±0.7	2.2±0.8	0.5
History of MI	45% (13)	60% (3)	0.5
Arterial hypertension	86% (25)	100% (5)	0.4
Diabetes	48% (14)	80% (4)	0.2
Heart rate (beats/minute)	66.4±11	68.2±13	0.8

AF = atrial fibrillation; GP = Ganglionated plexi; LA = left atrium; LVEF = left ventricular ejection fraction; MI = myocardial infarction; NYHA = New York Heart Association

Preoperative HR significantly influenced GP detection. Average preoperative HR was 66.8 (range: 49-88 beats/min.). All patients were on beta-blocker up to the day of the surgery. Two types of beta-blockers were used preoperatively: bisoprolol (5mg per day) and metoprolol (50 mg per day). Type and dose of beta-blocker was not changed at admission to the hospital. Type or dose of beta-blocker used preoperatively did not correspond with preoperative HR. According to anamnesis, all patients were on maximal dosage tolerated by them in terms of atrial blood pressure. Moreover, there was no statistically significant difference in preoperative HR between patients in sinus rhythm (63.9±16 beat/min.) and patients in AF (69.5±18 beat/min.; *P*=0.1). We found that preoperative HR exceeding 75 beats/min. increased the risk of negative GP detection in all tested areas (OR 7.3; 95% CI 2-25). In addition, preoperative HR of less than 70 beats/min. increased the probability of positive GP detection (OR 7; 95% CI 1.5-32). Similarly, in patients with preoperative HR of less than 60 beats/min., increased probability of positive GP detection was found (OR 5; 95% CI 1.6-15).

### Number of GP Detected

In patients with positive GP detection, a correlation between the number of GP detected and preoperative data was sought (Table 3). The number of GP detected correlated significantly only with preoperative resting HR, showing an inverse relationship (Figure 2). The cut-off value of preoperative HR for a significant difference between number of GP detected was found at 75 beats/min. Preoperative HR < 75 beats/min. correlated significantly with the total number of GP detected (r=-0.3516; *P*=0.04) and the number of right-sided GP detected (r=-0.4403; *P*=0.01), but not with the number of left-sided GP detected (r=-0.1716; *P*=0.3). The average number of total, left and right GP detected was significantly lower in patients with preoperative HR > 75 beats/min., both in the entire study population and after exclusion of patients with negative GP detection (Tables 4 and 5).

**Table 3:** Correlation between the number of GP detected and preoperative variables.

Parameter	Correlation factor	*P* value
Duration of AF (months)	0.0219	0.9
LA diameter (mm)	- 0.0142	0.9
Arterial hypertension	0.0359	0.8
EuroSCORE II (%)	0.0261	0.8
Diabetes	0.0681	0.7
LVEF (%)	0.0661	0.7
Age (years)	0.1025	0.5
History of MI	- 0.1174	0.5
NYHA class	- 0.2150	0.2
Syntax score	- 0.2937	0.09
Heart rate (beats/minute)	- 0.3509	0.04

AF = atrial fibrillation; LA = left atrium; LVEF = left ventricular ejection fraction; MI = myocardial infarction; NYHA = New York Heart Association

**Table 4:** Comparison of the average number of GP detected in relation to preoperative heart rate in the entire study population.

Number of GP detected	Group with HR < 75 (n=25)	Group with HR > 75 (n=9)	*P* value
Total GP detected	3.2±2.6	1.3±1	0.04
Left-side GP detected	1±0.7	0.6±0.7	0.3
Right-side GP detected	2.2±0.7	0.7±0.7	0.001

GP = Ganglionated plexi; HR = Heart rate

**Table 5:** Comparison of the average number of GP detected in relation to preoperative heart rate in patients with successful GP detection.

Number of GP detected	Group with HR < 75 (n=22)	Group with HR > 70 (n=7)	*P* value
Total GP detected	3.7±2.4	1.7±0.9	0.04
Left-side GP detected	1.8±1.4	0.7±0.7	0.4
Right-side GP detected	2.5±1.4	0.8±0.7	0.01

GP = Ganglionated plexi; HR = Heart rate

In patients with positive GP detection, there was no significant difference in average number of GP detected between the following groups: patients with persistent *versus* long-standing persistent AF (respectively: 2.7±1.3 *vs*. 4.1±3.4; *P*=0.1); diabetic *versus* non-diabetic patients (respectively: 3.7±2.8 *vs.* 2.7±1.5; *P*=0.2); patients with *versus* without previous myocardial infarction (respectively: 3±2.1 *vs.* 3.7±2.5; *P*=0.6); and patients with *versus* without arterial hypertension (respectively: 2.5±1.9 *vs*. 3.3±2.3; *P*=0.5).

### GP Detection and Arrhythmia Recurrence

There was one (2.9%) in-hospital death due to pneumonia and respiratory disorder. Study end-point was completed in 33 patients. In-hospital AF recurrence was observed in 14 (41%) patients and, in all of them, at least one electrical cardioversion was performed. At hospital discharge and 3 months after surgery, AF was recorded in 8 (24%) patients. None of the patients required pacemaker implantation. Combined study end-point (in-hospital AF recurrence requiring electrical cardioversion, AF at hospital discharge and after 3 months) occurred in 16 (48%) patients.

The number of GP detected correlated significantly with in-hospital AF recurrence requiring electrical cardioversion (r=0.3673; *P*=0.03) and with the number of electrical cardioversions performed (r=0.3844; *P*=0.02). In-hospital AF recurrence and the number of electrical cardioversions performed correlated significantly with the number of right-sided GP detected (r=0.3609; *P*=0.03 and r=0.3650; *P*=0.03, respectively), but not with the number of left-sided GP detected (r=0.2992; p=0.09 and r=0.3253; *P*=0.06, respectively). Average number of GP detected was significantly higher in patients with in-hospital AF recurrence requiring electrical cardioversion (3.8±3) in comparison to the group with stable sinus rhythm during in-hospital stay (2±1.3; *P*=0.02). In the group with in-hospital AF recurrence, mean number of right-sided GP detected was significantly higher compared to the rest of the population (2.4±1.7 *vs.* 1.3±1, respectively; *P*=0.02). Average number of left-sided GP detected did not differ between patients with in-hospital AF recurrence (1.3±1.5) and patients with stable sinus rhythm (0.6±0.8; *P*=0.08). In patients in whom 4 or more GP were detected, significantly increased risk of in-hospital AF recurrence was observed (OR 15; 95% CI 1.5-164; *P*=0.003).

## DISCUSSION

AF may be triggered and perpetuated by the autonomic nervous system (ANS) through the facilitation of premature atrial depolarization, shortening of the effective refractory period and an increased refractoriness heterogeneity of the atrial myocardium and the PVs^[4,7]^. Numerous studies have indicated that AF triggers and drivers (rotors, multiple wavelets) located in the PVs and posterior left atrial wall are generated by the ANS^[8,9]^. GP are formed from epicardial extensions of mediastinal nerves entering the heart. The highest density of cardiac GP is found in the fat pads surrounding the PV's entrance to the left atrium^[10]^. Those regions are the targets for GP detection and ablation during both surgical and transcatheter procedures.

Surgical GP ablation is a technique which is being applied as an adjunct strategy to the standard atrial fibrillation ablation procedure (MAZE III or PV isolation). Surgical GP ablation has been added to surgery due to promising data from transcatheter ablation studies^[11,12]^. Standard surgical GP ablation is based on GP detection. Vagal reflex is induced with high frequency (800 bpm) low voltage epicardial stimulation (5-10 V) of certain areas around the PVs ostia and the left atrial antrum. Epicardial mapping usually consists of applying current to 9 to 12 areas on each side. High frequency stimulation over GP results in rapid acetylcholine secretion. This signal is transmitted through atrial autonomic pathways to the sinus and atrio-ventricular nodes, resulting in transient bradycardia or an atrio-ventricular conduction block (via the vagal reflex). The epicardial ablation probe is then focally applied over the heart segment whose stimulation elicited such activity.

Several authors have reported different numbers of GP detected in different heart segments using this technique. Mehall et al.^[6]^ detected 0 to 10 active GP. In their cohort, a mean of 5 GP on the right side and 2.7 GP on the left side were ablated. The most common location of GP was found to be over the right superior PV and the left superior PV - in the area of the Marshall fold^[6]^. Kondo et al.^[5]^ reported an average number of 2.2 GP detected on the right and 0.4 on the left side. In this study population, GP detection was unsuccessful in 19% of the patients, and the most common location of detected GP was the area at the right inferior PV and between the right PVs.

It has been shown that the number and the location of detected left atrial GP is not fully consistent with the underlying anatomy^[13]^. Therefore, the GP detected during surgical procedures have been called "active ganglia". However, there is no proof that the undetectable ganglia are not physiologically active in those patients. Moreover, none of the anatomical studies have shown the absence of GP in areas where their detection was not successful during surgical procedures. Those findings may reveal a shortcoming of the detection technique. Thus far, there are no studies revealing the factors leading to positive GP detection during cardiac surgical procedures. In our study, the number of detected GP depended only on preoperative resting heart rate. A lower heart rate might be due to higher parasympathetic activity and thus increase the chance of a positive vagal reflex in those patients. Other factors (including age and type of AF) did not play a significant role in the success of GP detection.

The study showed significant correlation between the number of GP detected and early recurrence of AF after surgical ablation. Furthermore, we found that a higher number of GP detected was related to a higher risk of AF recurrence. Additionally, patients with a higher number of detected GP presented lower preoperative resting HR. These findings may support the thesis that, in patients with positive detection of GP, the density of ANS structures and concentration of neurotransmitter in left atrial fat pads (acetylcholine) is high. This may explain the easier induction of vagal reflex in that subgroup. However, undetected ganglia may secrete neurotransmitters postoperatively, lowering the threshold of early postoperative AF. It has been shown that postoperative AF after cardiac surgical procedures is related to ANS activity^[14]^. Eventually, lower preoperative HR may be a marker of parasympathetic predominance in this study population.

There is no general consensus on the long-term efficacy of GP ablation during surgical AF ablation procedures^[15]^. It is recommended as an additional strategy that might reduce early and mid-term AF recurrence^[2]^. However, the efficacy of surgical GP ablation may be underestimated due to the flaws of the detection technique. Our finding that the efficacy of GP detection was related only to preoperative HR may provide support for the shortcomings of the detection technique. For this reason, any attempt at evaluating the success of surgical GP ablation in decreasing AF recurrence is limited by imperfect GP detection. Thus, we suppose that incomplete epicardial denervation of the left atrium based on inadequate GP detection may limit the potential effectiveness of surgical autonomic modulation.

The findings of this study have resulted in a modification of the surgical technique used. We decided to perform total epicardial ablation of the left atrial fat pads regardless of the results of GP detection. The effectiveness of this approach is being evaluated in a prospective study.

## CONCLUSION

The number of detected GP correlates significantly with a lower preoperative heart rate. The success of GP detection and the number of GP detected remains unrelated to all other preoperative factors in patients undergoing surgical ablation of AF. Higher number of detected GP is associated with increased risk of early recurrence of AF after surgical ablation. The current GP detection technique based on vagal reflex induction resulted in the omission of fifteen percent of the study patients from left atrial autonomic denervation.

**Table:** 

Authors' roles & responsibilities	
GS	Conception and study design; realization of operations; analysis and/or data interpretation; statistical analysis; manuscript writing or critical review of its content; final manuscript approval
MMM	Conception and study design; analysis and/or data interpretation; statistical analysis; manuscript writing or critical review of its content; final manuscript approval
KK	Conception and study design; analysis and/or data interpretation; statistical analysis; manuscript writing or critical review of its content; final manuscript approval
JM	Conception and study design; analysis and/or data interpretation; statistical analysis; manuscript writing or critical review of its content; final manuscript approval
LG	Conception and study design; analysis and/or data interpretation; statistical analysis; manuscript writing or critical review of its content; final manuscript approval
AC	Conception and study design; analysis and/or data interpretation; statistical analysis; manuscript writing or critical review of its content; final manuscript approval
AS	Conception and study design; analysis and/or data interpretation; statistical analysis; manuscript writing or critical review of its content; final manuscript approval
